# Supplementary material for: Expression of Huntingtin and TDP-43 Derivatives in Fission Yeast Can Cause Both Beneficial and Toxic Effects
Source: Int J Mol Sci. 2022 Apr 1;23(7):3950. doi: 10.3390/ijms23073950 (PMC8999813; doi:10.3390/ijms23073950)
Supplement: Supplementary file 1 [file ijms-23-03950-s001.zip › ijms-1645956-supplementary.pdf]

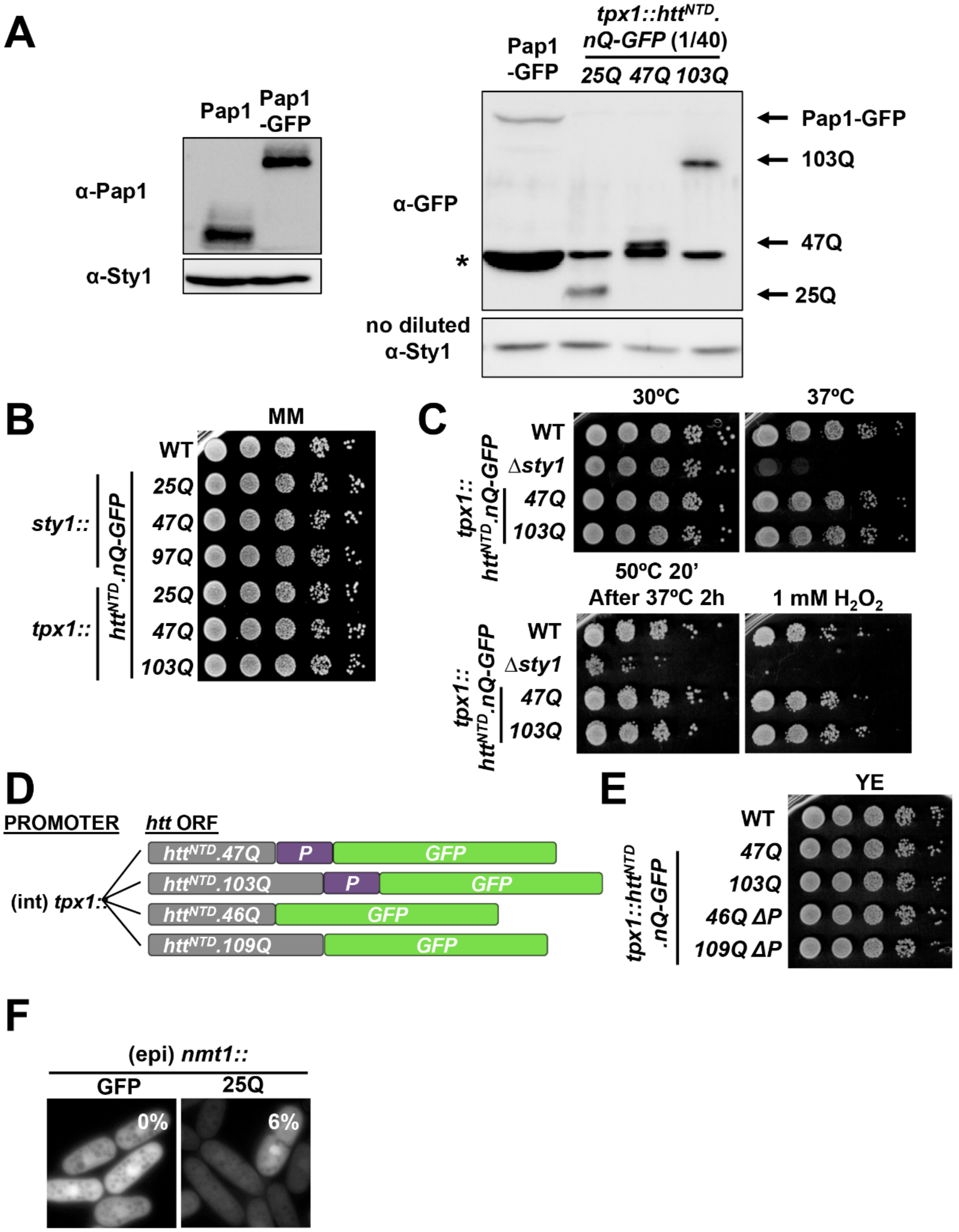

Figure S1. Expression of Htt derivatives in fission yeast (**A**) (Left panel) Endogenous Pap1 and endogenously tagged Pap1-GFP steady-state levels were determined by Western blot of native extracts from a wild-type strain (972) or a strain expressing Pap1-GFP (IC132). Sty1 was used as loading control. (Right panel) Endogenously tagged Pap1-GFP and *tpx1*-driven Htt^{NTD}.25Q-GFP, Htt^{NTD}.47Q-GFP and Htt^{NTD}.103Q-GFP steady-state levels determined by Western blot of non-diluted native extracts from a strain expressing Pap1-GFP (IC132) and of a 1/40 dilution of native extracts from strains expressing Htt^{NTD}.nQ-GFP (AB1.25Q, AB1.47Q and AB1.103Q). Non diluted extracts to determine Sty1 levels were used as loading control. (*) indicates a non-specific band detected by the polyclonal GFP antibody. (**B**) Wild-type (972) and strains expressing *tpx1*-driven Htt^{NTD}.nQ-GFP (AB1.25Q, AB1.47Q and AB1.103Q) or *sty1*-driven Htt^{NTD}.nQ-GFP (SB293.25Q, LM30.47Q and LM30.103Q) were serially diluted and spotted on solid MM plates. (**C**) Resistance to

different stresses. Wild-type (972), *Δsty1* (AV18) and strains expressing *tpx1*-driven Htt^{NTD}.nQ-GFP (AB1.47Q and AB1.103Q) growing logarithmically were either serially diluted and plated on YE5S plates at 30 °C, with and without 1mM H_2O_2, and at 37 °C or subjected to a 50 °C 20 min treatment preceded by 2h at 37 °C and then serially diluted and plated on YE5S plates. (**D**) Schematic representation of the Htt^{NTD}.nQ-GFP constructs expressed under the control of the integrative *tpx1* promoter. Gray box: N-terminal region of human HTT containing a stretch of 25, 47 or 103 glutamines; purple box: the proline-rich domain; green box: GFP. (**E**) Wild-type (972) and cells expressing *tpx1*-driven Htt^{NTD}.nQ-GFP (AB1.47Q and AB1.103Q), Htt^{NTD}.nQΔP-GFP (AB19.46Q and AB19.109Q) were serially diluted onto YE5S plates. (**F**) Fluorescence microscopy of cells expressing episomal GFP or Htt^{NTD}.25Q-GFP under the control of the *nmt1* promoter [HM123 transformed with p690.3x (GFP) or with p659.25Q.3x (Htt^{NTD}.25Q-GFP)] grown in MM. The percentage of cells with aggregates is indicated. *N* = 50 for 25Q construct.

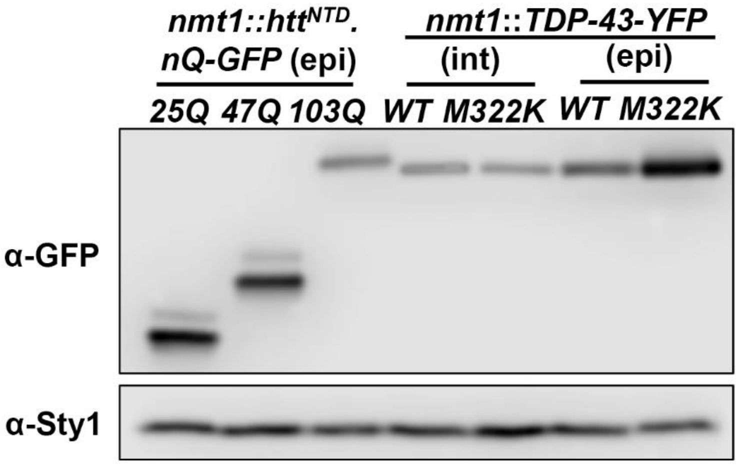

Figure S2. Expression of TDP-43 derivatives in fission yeast. Steady-state levels of Htt^{NTD}.nQ-GFP and TDP-43-YFP determined by Western blot of TCA extracts from strains expressing episomal *nmt1*-driven Htt^{NTD}.nQ-GFP (HM123 transformed with p659.25Q.3x, p659.47Q.3x and p659.103Q.3x), integrative *nmt1*-driven TDP-43-YFP and TDP-43.M322K-YFP (LM218 and LM218.M322K) and episomal *nmt1*-driven TDP-43-YFP and TDP-43.M322K-YFP [HM123 transformed with p660.3x (*WT*) and p660.M322K.3x (*M322K*)] and grown in MM. Sty1 was used as loading control.

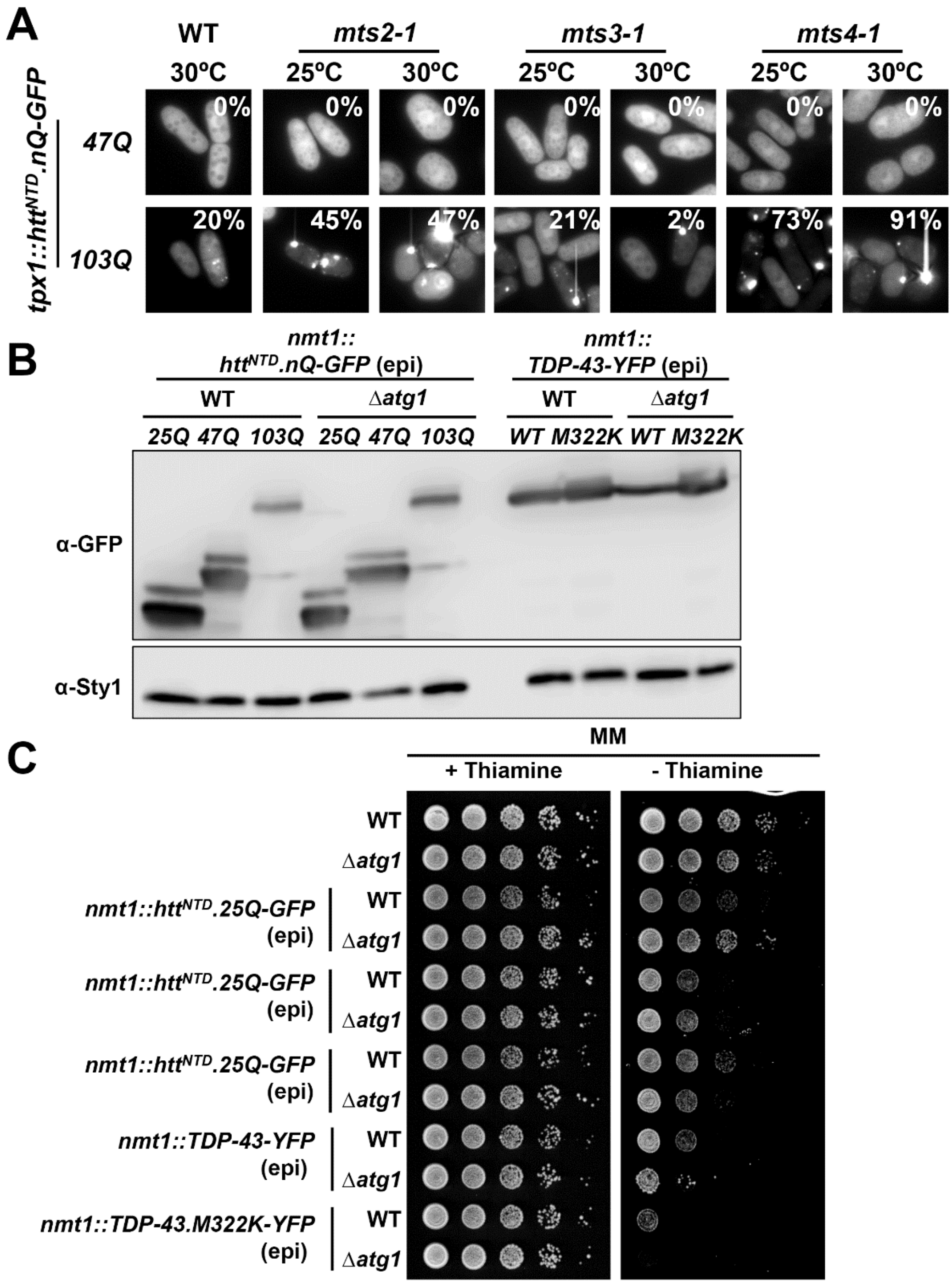

Figure S3. Htt^{NTD}.nQ-GFP and TDP-43-YFP are not degraded by the UPS or autophagy. (**A**) Fluorescence microscopy of wild-type, *mts2-1*, *mts3-1* and *mts4-1* strains expressing *tpx1*-driven Htt^{NTD}.nQ-GFP (WT: AB1.47Q, AB1.103Q; *mts2-1*: LM236.47Q and LM236.103Q; *mts3-1*: LM237.47Q and LM237.103Q; *mts4-1*: LM238.47Q and LM238.103Q) and growing at 25 °C or 30 °C. The percentage of cells with aggregates is indicated. *N* =50, 100, 100, 100, 100, 100 and 100, for 103Q constructs from left to right. (**B**) Steady-state levels of Htt^{NTD}.nQ-GFP, TDP43-YFP and TDP43.M322K-YFP determined by Western blot of TCA extracts from wild-type and *Δatg1* cells grown in MM and expressing episomal *nmt1*-driven Htt^{NTD}.nQ-GFP (HM123 and SK1 transformed with p659.25Q.3x, p659.47Q.3x and p659.103Q.3x) or expressing episomal *nmt1*-driven TDP-43-YFP and TDP-43.M322K-YFP (HM123 and SK1 transformed with p660.3x (*WT*) and p660.M322K.3x (*M322K*). (**C**) Strains as in B were serially diluted and spotted onto MM plates as in Figure 1C.

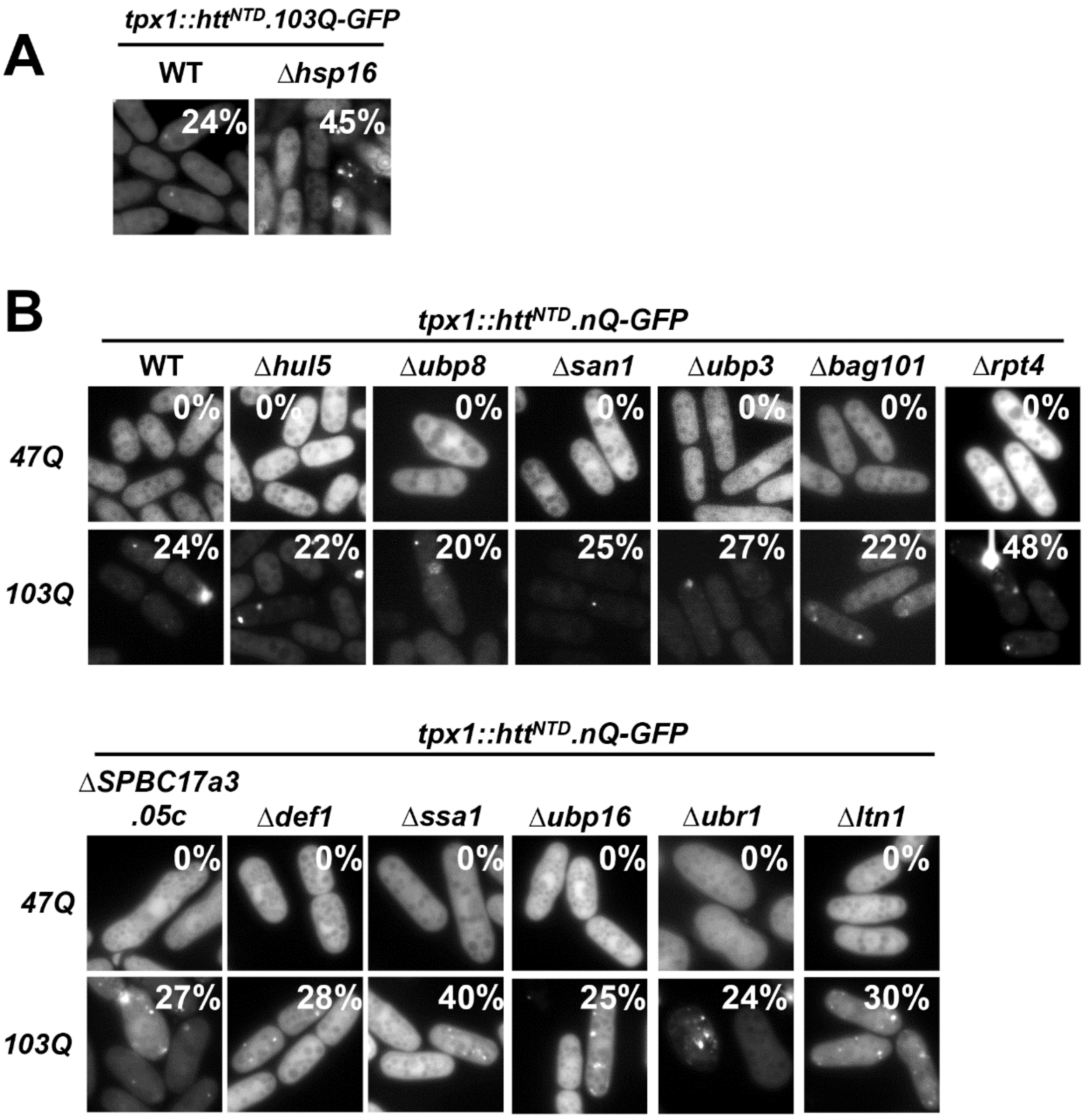

Figure S4. Only few components of the PQC system are involved in Htt^{NTD}.nQ-GFP aggregation. (**A**) Fluorescence microscopy of strains expressing Htt^{NTD}.103Q-GFP under the control of constitutive *tpx1* promoter in wild-type (AB1.103Q) and *Δhsp16* (LM52.103Q) strains. The percentage of cells with aggregates is indicated. *N* = 56 and *N* = 100 for WT and *Δhsp16* respectively. (**B**) Fluorescence microscopy of strains expressing Htt^{NTD}.nQ-GFP under the control of constitutive *tpx1* promoter in wild-type (AB1.47Q and AB1.103Q), *Δhul5* (LM20.47Q and LM20.103Q), *Δubp8* (LM24.47Q and LM24.103Q), *Δsan1* (LM10.47Q and LM10.103Q), Δ*ubp3* (LM13.47Q and LM13.103Q), *Δbag101* (LM27.47Q and LM27.103Q), *Δrpt4* (LM21.47Q and LM21.103Q), *ΔSPBC17A3.05c* (AB7.47Q and AB7.103Q), *Δdef1* (AB8.47Q and AB8.103Q), *Δssa1* (AB9.47Q and AB9.103Q), *Δubp16* (LM25.47Q and LM25.103Q), *Δubr1* (LM235.47Q and LM235.103Q), and *Δltn1* (LM247.47Q and LM247.103Q). The percentage of cells with aggregates is indicated. *N* =54, 72, 51, 59, 52, 127 and 58 for 103Q constructs in upper panel from left to right. *N* = 51, 68, 74, 52, 100 and 85 for 103Q constructs in lower panel from left to right.

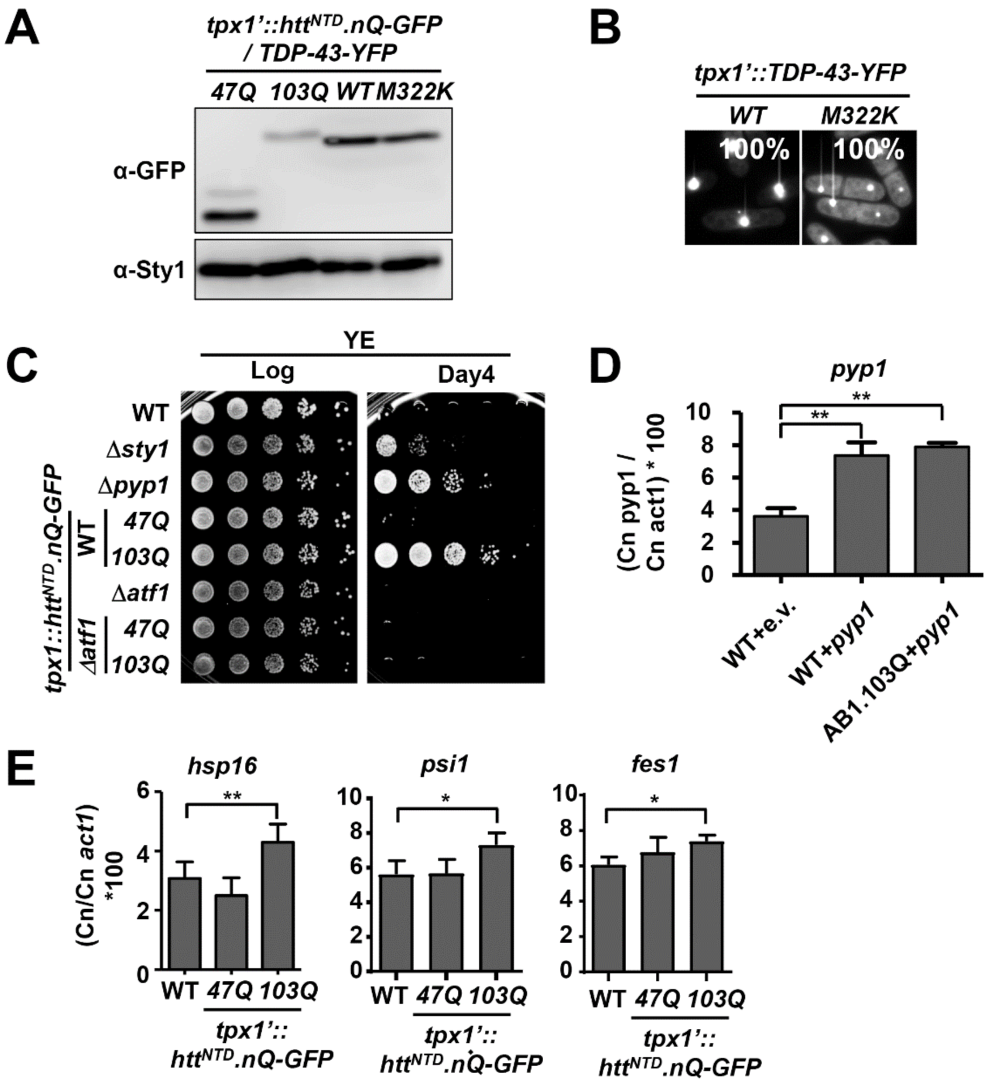

Figure S5. Expression of *tpx1* promoter-driven 103Q promotes lifespan extension. (**A**) Steady-state levels of and Htt^{NTD}.nQ-GFP and TDP-43-YFP determined by Western blot of TCA extracts from strains expressing *tpx1*-driven Htt^{NTD}.nQ-GFP (AB1.47Q and AB1.103Q), or *tpx1*-driven TDP-43-YFP and TDP43.M233K-YFP (LM210 (*WT*) and LM210.M322K (*M322K*). Sty1 was used as loading control. (**B**) Fluorescence microscopy of strains expressing TDP-43-YFP under the control of the constitutive *tpx1* promoter (LM210 (*WT*) and, LM210.M322K (*M322K*). The percentage of cells with aggregates is indicated. *N* = 50. (**C**) Wild-type (972), *Δsty1* (AV18), *Δpyp1* (EP48), wild-type strains expressing *tpx1*-driven Htt^{NTD}.nQ-GFP (AB1.47Q and AB1.103Q), *Δatf1* (MS98) and *Δatf1* strains expressing *tpx1*-driven Htt^{NTD}.nQ-GFP (LM23.47Q and LM23.103Q) were grown in YE5S for 4 days. Cultures from logarithmic (Log) or stationary phase (Day 4) were serially diluted and spotted onto YE5S plates. (**D**) The mRNA levels of *pyp1* were determined by RT-qPCR in a wild-type strain with an empty vector (SB472), a wild-type strain with an extra copy of *pyp1* under the control of its own promoter (SB706) and a strain expressing *tpx1*-driven Htt^{NTD}.103Q-GFP with an extra copy of *pyp1* under the control of its own promoter (SB707). Data are expressed as the mRNA copy number (Cn) relative to actin Cn and represent the average of three biological replicates. Error bars represent SD. Statistical significance was calculated between the indicated samples with an unpaired Student's t test and 95% confidence level with p values of 0.01. (**E**) The mRNA levels of the Hsf1-dependent genes *hsp16*, *psi1* and *fes1* were determined by RT-qPCR in a wild-type and in strains expressing tpx1-driven Htt^{NTD}.nQ-GFP (AB1.47Q and AB1.103Q). Data are expressed as the mRNA copy number (Cn) relative to actin Cn and represent the average of at least three biological replicates. Error bars represent SD. Statistical significance was calculated between the indicated samples with an unpaired Student's t test and 95% confidence level with * $p = 0.05$ and * $p = 0.01$.

Table S1. Strains used in this study.

Strain	Genotype	Origin
972	h^-	[1]
AB1.25Q	h^- *tpx1::htt*^{NTD}*.25Q-GFP::leu1*	This work
AB1.47Q	h^- *tpx1::htt*^{NTD}*.47Q-GFP::leu1*	This work
AB1.103Q	h^- *tpx1::htt*^{NTD}*.103Q-GFP::leu1*	This work
AB2.47Q	h^- *hsp104::kanMX6 tpx1::htt*^{NTD}*.47Q-GFP::leu1*	This work
AB2.103Q	h^- *hsp104::kanMX6 tpx1::htt*^{NTD}*.103Q-GFP::leu1*	This work
AB6.103Q	h^- *leu1-32 tpx1::htt*^{NTD}*.Q103-GFP::leu1 leu1::natMX6*	This work
AB7.47Q	h^- *SPBC17A3.05c::kanMX6 tpx1::htt*^{NTD}*.47Q-GFP::leu1*	This work
AB8.47Q	h^- *def1::kanMX6 tpx1::htt*^{NTD}*.47Q-GFP::leu1*	This work
AB9.47Q	h^- *ssa1::kanMX6 tpx1::htt*^{NTD}*.47Q-GFP::leu1*	This work
AB19.46QΔP	h^- *tpx1::htt*^{NTD}*.46QΔP-GFP::leu1*	This work
AB19.109QΔP	*tpx1::htt*^{NTD}*.109QΔP-GFP::leu1*	This work
AV18	h^- *sty1::kanMX6*	[2]
EP48	h^+ *pyp1::natMX6*	This work
HM123	h^- *leu1-32*	[3]
LM10.47Q	h^+ *san1::kanMX6 tpx1::htt*^{NTD}*.47Q-GFP::leu1 ade6-M210 ura4-D18*	This work
LM10.103Q	h^+ *san1::kanMX6 tpx1::htt*^{NTD}*.103Q-GFP::leu1 ade6-M210 ura4-D18*	This work
LM13.47Q	h^+ *ubp3::kanMX6 tpx1::htt*^{NTD}*.47Q-GFP::leu1 ade6-M210 ura4-D18*	This work
LM13.103Q	h^+ *ubp3::kanMX6 tpx1::htt*^{NTD}*.103Q-GFP::leu1 ade6-M210 ura4-D18*	This work
LM18.47Q	h^- *mas5::kanMX6 sty1::htt*^{NTD}*.47Q-GFP::leu1*	This work
LM18.103Q	h^- *mas5::kanMX6 sty1::htt*^{NTD}*.103Q-GFP::leu1*	This work
LM20.47Q	h^+ *hul5::kanMX6 tpx1::htt*^{NTD}*.47Q-GFP::leu1 ade6-M210 ura4-D18*	This work
LM20.103Q	h^+ *hul5::kanMX6 tpx1::htt*^{NTD}*.103Q-GFP::leu1 ade6-M210 ura4-D18*	This work
LM21.47Q	h^+ *rpt4::kanMX6 tpx1::htt*^{NTD}*.47Q-GFP::leu1 ade6-M210 ura4-D18*	This work
LM21.103Q	h^+ *rpt4::kanMX6 tpx1::htt*^{NTD}*.103Q-GFP::leu1 ade6-M210 ura4-D18*	This work
LM23.47Q	h^- *atf1::kanMX6 tpx1::htt*^{NTD}*.47Q-GFP::leu1*	This work
LM23.103Q	h^- *atf1::kanMX6 tpx1::htt*^{NTD}*.103Q-GFP::leu1*	This work
LM24.47Q	h^+ *ubp8::kanMX6 tpx1::htt*^{NTD}*.47Q-GFP::leu1 ade6-M210 ura4-D18*	This work
LM24.103Q	h^+ *ubp8::kanMX6 tpx1::htt*^{NTD}*.103Q-GFP::leu1 ade6-M210 ura4-D18*	This work
LM25.47Q	h^+ *ubp16::kanMX6 tpx1::htt*^{NTD}*.47Q-GFP::leu1 ade6-M210 ura4-D18*	This work
LM25.103Q	h^+ *ubp16::kanMX6 tpx1::htt*^{NTD}*.103Q-GFP::leu1 ade6-M210 ura4-D18*	This work
LM27.47Q	h^+ *bag101::kanMX6 tpx1::htt*^{NTD}*.47Q-GFP::leu1 ade6-M210 ura4-D18*	This work
LM27.103Q	h^+ *bag101::kanMX6 tpx1::htt*^{NTD}*.103Q-GFP::leu1 ade6-M210 ura4-D18*	This work
LM30.47Q	h^- *sty1::htt*^{NTD}*.47Q-GFP::leu1*	This work
LM30.103Q	h^- *sty1::htt*^{NTD}*.103Q-GFP::leu1*	This work
LM52.103Q	h^- *hsp16::kanMX6 tpx1::htt*^{NTD}*.103Q-GFP::leu1*	This work
LM208.25Q	h^- *nmt1::htt*^{NTD}*.25Q-GFP::leu1*	This work
LM208.47Q	h^- *nmt1::htt*^{NTD}*.47Q-GFP::leu1*	This work
LM208.103Q	h^- *nmt1::htt*^{NTD}*.103Q-GFP::leu1*	This work
LM210	h^- *tpx1::tdp-43-YFP::leu1*	This work
LM210.M322K	h^- *tpx1::tdp-43.M322K-YFP::leu1*	This work
LM217.47Q	h^- *sty1::mCherry-mas5::leu1 tpx1::htt*^{NTD}*.47Q-GFP::leu1::kanMX6*	This work
LM217.103Q	h^- *sty1::mCherry-mas5::leu1 tpx1::htt*^{NTD}*.103Q-GFP::leu1::kanMX6*	This work
LM218	h^- *nmt1::tdp-43-YFP::leu1*	This work
LM218.M322K	h^- *nmt1::tdp-43.M322K-YFP::leu1*	This work
LM219	h^- *hsp104::kanMX6 nmt1::TDP-43-YFP::leu1*	This work
LM219.M322K	h^- *hsp104::kanMX6 nmt1′::TDP-43.M322K-YFP::leu1*	This work
LM225	h^- *mas5::kanMX6 nmt1::TDP-43-YFP::leu1*	This work
LM225.M322K	h^- *mas5::kanMX6 nmt1::TDP-43.M322K-YFP::leu1*	This work
LM233	h^- *nmt41::tdp-43-YFP::leu1*	This work
LM233.M322K	h^- *nmt41::tdp-43.M322K-YFP::leu1*	This work
LM235.47Q	h^+ *ubr1::kanMX6 tpx1′::htt*^{NTD}*.47Q-GFP::leu1*	This work
LM235.103Q	h^+ *ubr1::kanMX6 tpx1′::htt*^{NTD}*.103Q-GFP::leu1*	This work
LM236.47Q	h^- *mts2-1 tpx1::htt*^{NTD}*.47Q-GFP::leu1*	This work
LM236.103Q	h^- *mts2-1 tpx1::htt*^{NTD}*.103Q-GFP::leu1*	This work
LM237.47Q	h^- *mts3-1 tpx1::htt*^{NTD}*.47Q-GFP::leu1*	This work

LM237.103Q	h^- mts3-1 tpx1::htt^{NTD}.103Q-GFP::leu1	This work
LM238.47Q	h^- mts4-1 tpx1::htt^{NTD}.47Q-GFP::leu1	This work
LM238.103Q	h^- mts4-1 tpx1::htt^{NTD}.103Q-GFP::leu1	This work
MS98	h^- atf1::natMX	[4]
RB44	h- pgk1-GFP::natMX6	Corral-Ramos 2021
SB293.25Q	h^- sty1::htt^{NTD}.25Q-GFP::leu1 ura4-D18	This work
SB413.47Q	h^- mas5::kanMX6 tpx1′::htt^{NTD}.47Q-GFP::leu1	This work
SB413.103Q	h^- mas5::kanMX6 tpx1′::htt^{NTD}.103Q-GFP::leu1	This work
SG287	h^+ hsp104::natMX6	This work
SK1	h^- atg1::ura4+ ura4-C190T leu1-32	[5]
BY4741	MATα his3Δ1 leu2Δ0 met15Δ0 ura3Δ0	[6]

Table S2. Plasmids used in this study.

Plasmid	Genotype	Origin
pRS426-GFP	GAL1::GFP::ura3	Addgene
pRS416-TDP-43-YFP	GAL1::TDP-43-YFP::ura3	[7]
pRS416-TDP-43. M322K-YFP	GAL1::TDP-43.M322K-YFP::ura3	[8]
p426/PQ25	GPD:: htt^{NTD}.25Q-GFP::ura3	[9]
p426/PQ47	GPD:: htt^{NTD}.47Q-GFP::ura3	[9]
p426/PQ103	GPD:: htt^{NTD}.103Q-GFP::ura3	[9]
p499.25Q	sty1::htt^{NTD}.25Q-GFP::leu1	This work
p499.47Q	sty1::htt^{NTD}.47Q-GFP::leu1	This work
p499.103Q	sty1::htt^{NTD}.103Q-GFP::leu1	This work
p503.25Q.	tpx1::htt^{NTD}.25Q-GFP::leu1	This work
p503.47Q	tpx1::htt^{NTD}.47Q-GFP::leu1	This work
p503.103Q	tpx1::htt^{NTD}.103Q-GFP::leu1	This work
p520.41x	nmt41::hsp104::leu1 (episomal)	This work
p414GPD.47Q	GPD:: htt^{NTD}.47QΔP-GFP	[10]
p414GPD.103Q	GPD:: htt^{NTD}.103QΔP-GFP	[10]
p521.46QΔP	tpx1::htt^{NTD}.46QΔP-GFP::leu1	This work
p521.109QΔP	tpx1::htt^{NTD}.109ΔP-GFP::leu1	This work
p659.25Q.3x	nmt1::htt^{NTD}.25Q-GFP::leu1 (episomal)	This work
p659.47Q.3x	nmt1::htt^{NTD}.47Q-GFP::leu1 (episomal)	This work
p659.103Q.3x	nmt1::htt^{NTD}.103Q-GFP::leu1 (episomal)	This work
p660.3x	nmt1::tdp-43-YFP::leu1 (episomal)	This work
p660.M322K.3x	nmt1::tdp-43.M322K-YFP::leu1 (episomal)	This work
p660.41x	nmt41::tdp-43-YFP::leu1 (episomal)	This work
p660.M322K.41x	nmt41::tdp-43.M322K-YFP::leu1 (episomal)	This work
p688.25Q.3x′	nmt1::htt^{NTD}.25Q-GFP::leu1	This work
p688.47Q.3x′	nmt1::htt^{NTD}.47Q-GFP::leu1	This work
p688.103Q.3x′	nmt1::htt^{NTD}.103Q-GFP::leu1	This work
p689	tpx1::tdp-43-YFP::leu1	This work
p689.M322K	tpx1::tdp-43.M322K-YFP::leu1	This work
p690.3x	nmt1::GFP::leu1 (episomal)	This work
p710.3x′	nmt1::tdp-43-YFP::leu1	This work
p710.M322K.3x′	nmt1::tdp-43.M322K-YFP::leu1	This work
p710.41x′	nmt41::tdp-43-YFP::leu1	This work
p710.M322K.41x′	nmt41::tdp-43.M322K-YFP::leu1	This work
p723.47Q	tpx1::htt^{NTD}.47Q-GFP::kanMX6	This work
p723.103Q	tpx1::htt^{NTD}.103Q-GFP::kanMX6	This work
p819′	pyp1::pyp1::hphMX6::leu1	This work

Table S3. % of cells with Htt or TDP-43 aggregates in different genetic backgrounds.

Medium	Strain	Promoter	Htt.25Q	Htt.47Q	Htt.103Q	TDP-43	TDP-43 M322K
MM	WT	*nmt41 (int)*	-	-	-	100 (nuc)	0
		nmt41 (epi)	-	-	-	100 (nuc)	100 (cyt)
		sty1	0	0	0	-	-
		tpx1	0	0	60	100 (nuc)	100 (cyt)
		nmt1 (int)	0	0	30	100 (nuc)	100 (cyt)
		nmt1 (epi)	6	50	20	100 (nuc/ cit)	100 (cyt)
	Δmas5	*nmt1 (int)*	-	-	-	100 (nuc)	100 (cyt)
	Δhsp104	*nmt1 (int)*	-	-	-	100 (nuc)	100 (cyt)
YE5S	WT	*sty1*	0	0	0	-	-
		tpx1	0	0	24	-	-
	Δmas5	*sty1*	-	0	24	-	-
		tpx1	-	27	98	-	-
	Δhsp104	*tpx1*	-	0	0	-	-
	O/E hsp104	*tpx1*	-	-	0	-	-
	Δhsp16	*tpx1*	-	0	45	-	-
	Δhul5	*tpx1*	-	0	22	-	-
	Δubp8	*tpx1*	-	0	20	-	-
	Δsan1	*tpx1*	-	0	25	-	-
	Δubp3	*tpx1*	-	0	27	-	-
	Δbag101	*tpx1*	-	0	22	-	-
	Δrpt4	*tpx1*	-	0	48	-	-
	ΔSPBC17a3 .05c	*tpx1*	-	0	27	-	-
	Δdef1	*tpx1*	-	0	28	-	-
	Δssa1	*tpx1*	-	0	40	-	-
	Δubp16	*tpx1*	-	0	25	-	-
	Δubr1	*tpx1*	-	0	24	-	-
	Δltn1	*tpx1*	-	0	30	-	-
	mts2-1 25°C	*tpx1*	-	0	45	-	-
	mts2-1 30°C	*tpx1*	-	0	47	-	-
	mts3-1 25°C	*tpx1*	-	0	21	-	-
	mts3-1 30°C	*tpx1*	-	0	2	-	-
	mts4-1 25°C	*tpx1*	-	0	73	-	-
	mts4-1 30°C	*tpx1*	-	0	91	-	-

The medium used is indicated. The temperature-sensitive mutants were analyzed at the permissive (25°C) and restrictive (30°C) temperatures. The nuclear of cytoplasmic localization of the TDP-43 aggregates is indicated.

References

1. Leupold, U., Genetical methods for *Schizosaccharomyces pombe*. *Methods Cell Physiol.* **1970,** 4, 169-177.
2. Zuin, A.; Vivancos, A. P.; Sanso, M.; Takatsume, Y.; Ayte, J.; Inoue, Y.; Hidalgo, E., The glycolytic metabolite methylglyoxal activates Pap1 and Sty1 stress responses in Schizosaccharomyces pombe. *J Biol Chem* **2005,** 280, (44), 36708-13.
3. Moreno, S.; Klar, A.; Nurse, P., Molecular genetic analysis of fission yeast Schizosaccharomyces pombe. *Methods Enzymol.* **1991,** 194, 795-823.
4. Fernandez-Vazquez, J.; Vargas-Perez, I.; Sanso, M.; Buhne, K.; Carmona, M.; Paulo, E.; Hermand, D.; Rodriguez-Gabriel, M.; Ayte, J.; Leidel, S.; Hidalgo, E., Modification of tRNA(Lys) UUU by elongator is essential for efficient translation of stress mRNAs. *PLoS Genet* **2013,** 9, (7), e1003647.
5. Mukaiyama, H.; Kajiwara, S.; Hosomi, A.; Giga-Hama, Y.; Tanaka, N.; Nakamura, T.; Takegawa, K., Autophagy-deficient Schizosaccharomyces pombe mutants undergo partial sporulation during nitrogen starvation. *Microbiology* **2009,** 155, (Pt 12), 3816-26.
6. Brachmann, C. B.; Davies, A.; Cost, G. J.; Caputo, E.; Li, J.; Hieter, P.; Boeke, J. D., Designer deletion strains derived from Saccharomyces cerevisiae S288C: a useful set of strains and plasmids for PCR-mediated gene disruption and other applications. *Yeast* **1998,** 14, (2), 115-32.

7. Johnson, B. S.; Snead, D.; Lee, J. J.; McCaffery, J. M.; Shorter, J.; Gitler, A. D., TDP-43 is intrinsically aggregation-prone, and amyotrophic lateral sclerosis-linked mutations accelerate aggregation and increase toxicity. *J Biol Chem* **2009,** 284, (30), 20329-39.
8. Bolognesi, B.; Faure, A. J.; Seuma, M.; Schmiedel, J. M.; Tartaglia, G. G.; Lehner, B., The mutational landscape of a prion-like domain. *Nat Commun* **2019,** 10, (1), 4162.
9. Krobitsch, S.; Lindquist, S., Aggregation of huntingtin in yeast varies with the length of the polyglutamine expansion and the expression of chaperone proteins. *Proc Natl Acad Sci U S A* **2000,** 97, (4), 1589-94.
10. Dehay, B.; Bertolotti, A., Critical role of the proline-rich region in Huntingtin for aggregation and cytotoxicity in yeast. *J Biol Chem* **2006,** 281, (47), 35608-15.
